# Can Nanofluidic Chemical Release Enable Fast, High Resolution Neurotransmitter-Based Neurostimulation?

**DOI:** 10.3389/fnins.2016.00138

**Published:** 2016-03-31

**Authors:** Peter D. Jones, Martin Stelzle

**Affiliations:** BioMEMS & Sensors, Natural and Medical Sciences Institute at the University of TübingenReutlingen, Germany

**Keywords:** nanofluidic, nanopore, microfluidic, neurotransmitter, neurotransmission, chemical neuroprosthesis, hydrophobic gating, artificial synapse

## Abstract

Artificial chemical stimulation could provide improvements over electrical neurostimulation. Physiological neurotransmission between neurons relies on the nanoscale release and propagation of specific chemical signals to spatially-localized receptors. Current knowledge of nanoscale fluid dynamics and nanofluidic technology allows us to envision artificial mechanisms to achieve fast, high resolution neurotransmitter release. Substantial technological development is required to reach this goal. Nanofluidic technology—rather than microfluidic—will be necessary; this should come as no surprise given the nanofluidic nature of neurotransmission. This perspective reviews the state of the art of high resolution electrical neuroprostheses and their anticipated limitations. Chemical release rates from nanopores are compared to rates achieved at synapses and with iontophoresis. A review of microfluidic technology justifies the analysis that microfluidic control of chemical release would be insufficient. Novel nanofluidic mechanisms are discussed, and we propose that hydrophobic gating may allow control of chemical release suitable for mimicking neurotransmission. The limited understanding of hydrophobic gating in artificial nanopores and the challenges of fabrication and large-scale integration of nanofluidic components are emphasized. Development of suitable nanofluidic technology will require dedicated, long-term efforts over many years.

## Introduction

Neuroprostheses are becoming increasingly important for treatment of neurological disorders (Borton et al., 2013). Cochlear implants have restored hearing to hundreds of thousands of patients (Shannon, 2012). Deep brain stimulation has helped more than 100,000 patients suffering from Parkinson's disease and may treat numerous additional diseases (Lozano and Lipsman, 2013). Retinal prostheses restore basic visual percepts in patients with previously-untreatable conditions such as retinitis pigmentosa (Zrenner, 2013). Stimulation of the somatosensory cortex has been proposed to restore touch and proprioception in patients with prosthetic limbs (Bensmaia, 2015); a first demonstration in humans was recently reported and is expected to be published soon (Sanchez, 2015).

Current neuroprostheses address the nervous system by electrical stimulation. Other potential modalities include optical and chemical stimulation. A clinical trial investigating optogenetic treatment of retinitis pigmentosa in humans has begun (RetroSense Therapeutics, 2015; Bourzac, 2016), but challenges of low photosensitivity and targeting of specific retinal cells must be overcome and ethical issues and safety of human genetic modification must be addressed (Barrett et al., 2014). Chemical stimulation has been proposed (Iezzi and Finlayson, 2011) and supported by preliminary experiments (Finlayson and Iezzi, 2010; Inayat et al., 2014). Although, implantable drug delivery systems have been used clinically for decades (Penn and Kroin, 1985) and neural probes capable of drug delivery have been demonstrated (Frey et al., 2011; Altuna et al., 2013; Pongrácz et al., 2013), the distinct concept of *functional* chemical stimulation capable of mimicking neurotransmission remains inaccessible. The absence of suitable technology is the primary roadblock, which will be discussed in more detail below.

### Neuronal signaling at chemical synapses

Neurons communicate with each other by the release of chemical neurotransmitters from the axonal terminal of presynaptic cells into the synaptic cleft (some neurons also use electrical synapses) (Purves et al., 2004). Release occurs by exocytosis of neurotransmitter-containing vesicles, triggered by an influx of calcium ions through voltage-gated channels which open upon depolarization of the axonal terminal by action potentials. Recognition of these chemical signals by specific, spatially-localized receptors in the postsynaptic membrane causes postsynaptic neurons to change their transmembrane potential in spatially-restricted areas, such as the synaptic cleft. Local changes of transmembrane potentials propagate across the cellular membrane, allowing integration of multiple synaptic inputs, and triggering successive neurotransmitter release to downstream neurons.

The specificity of chemical signaling relies on recognition of neurotransmitter by postsynaptic receptors. Prevention of continued stimulation of postsynaptic neurons requires removal of neurotransmitters from the extracellular volume. Most neurotransmitters are recycled by uptake into presynaptic neurons or degraded enzymatically.

Physiological concentrations of neurotransmitters cover at least seven orders of magnitude, from nanomolar to more than 100 mM (Featherstone, 2010). However, quantification of both synaptic and ambient extracellular concentrations is challenging (Scimemi and Beato, 2009; Sun et al., 2014). Peak synaptic glutamate concentrations are estimated to be in the low millimolar range (0.5–5 mM) (Featherstone, 2010). Nanomolar concentrations of ambient extracellular glutamate were predicted (Zerangue and Kavanaugh, 1996) and measured (Herman and Jahr, 2007). Other measurements revealed a wide range (25 nM to 10 μM), but higher concentrations are believed to result from damage due to the measurement technique (Sun et al., 2014).

### Electrical stimulation

Electrical neurostimulation depolarizes cells by extracellular voltage gradients generated by the spread of current injected into tissue by electrodes (Durand, 2000). Efforts have been made to target specific membrane areas or neuronal processes to modulate neuronal signaling by triggering or inhibiting release of neurotransmitters. However, electrical neurostimulation does not mimic physiological neurotransmission as the stimulating electric fields produce unspecific polarization of cells and act in large volumes compared to the size of synapses and neuronal processes. Electric fields may stimulate any cellular structures, depending on their spatial arrangement with nearby electrodes. This contrasts with neurotransmission, in which receptors on local areas of cell membranes respond to specific chemical signals. The main advantage of electrical stimulation compared to chemical stimulation is that the electrical field can be switched within microseconds at arbitrary locations. Technology to inject chemical signals with similar precision and speed does not exist, and chemical signals must rely on other mechanisms for removal.

Neuroprostheses rely on technology adapted from the microelectronics industry. Although modern nanofabrication achieves sub-10-nm resolution, extracellular neurostimulation electrodes maintain dimensions of tens of micrometers. The challenge of injecting sufficient current to excite neurons must be met while avoiding high voltages and dangerous side reactions (Merrill et al., 2005).

Preclinical experiments predicted a resolution limit of tens of micrometers for electrical stimulation of the retina (Stett et al., 2007). Intracortical microstimulation excites neurons hundreds of micrometers away from electrodes, although producing distinct percepts with closely-spaced electrodes has not been investigated (Bensmaia, 2015). Current retinal prostheses approach the predicted limits, with the Alpha IMS prosthesis (Retina Implant AG, Reutlingen, Germany) having 1500 electrodes with 70 μm pitch. The best artificial visual acuity demonstrated until now is 0.037 (Snellen acuity of 20/546), corresponding to a spatial resolution of 126 μm on the retina (Stingl et al., 2013). In comparison, visual acuity achieved by the fovea of healthy human retinas relies on photoreceptor cells with a pitch of ~5 μm (Hirsch and Curcio, 1989).

New electrode materials such as conducting polymers stimulate at lower voltages (Gerwig et al., 2012; Samba et al., 2015), which may enable the safe operation of smaller electrodes. Investigations with complex electrode configurations (Lorach et al., 2015) and guidance of neurons into features on artificial devices (Adekunle et al., 2015) are attempting to address these issues. However, a recent review emphasizes that electrical stimulation is not expected to restore visual acuity approaching normal vision (Eiber et al., 2013).

Recently, electrical recording and stimulation of single cells has been achieved by nanoscale electrodes (Angle et al., 2015), which may penetrate the cell membrane (Qing et al., 2013) or be engulfed (Hai et al., 2010). Reproduction of these results *in vivo* will be necessary to evaluate their potential for use in neuroprostheses. Although promising for high resolution stimulation, such methods would continue to rely on unphysiological mechanisms.

### Progress toward chemical stimulation

Researchers have long attempted to mimic neurotransmission. Neurotransmitter release from micropipettes by pressure injection or iontophoresis is an important technique for neurochemical investigations (Lalley, 1999). Such methods confirmed that delivery of glutamate produces physiological responses in retinas, supporting the concept of chemical-based prostheses (Finlayson and Iezzi, 2010; Inayat et al., 2014). Communicating in the extracellular language of neurons allows chemical stimulation to mimic real synaptic neurotransmission (Murnick et al., 2002; Finlayson and Iezzi, 2010; Iezzi and Finlayson, 2011; Inayat et al., 2014).

As with electrical neuroprostheses, advanced chemical stimulation devices would require dense integration by micro- or nanofabrication, which cannot be realized with micropipettes. Microfluidic devices for parallel chemical stimulation from an array of sites have been demonstrated, which release chemical stimuli through apertures addressed by buried channels (Peterman et al., 2004; Scott et al., 2013). Microfluidic devices were also proposed following pipette-based investigations (Inayat et al., 2014). A critical shortcoming of microfluidic devices is their failure to suppress leakage by diffusion. Although diffusion can be countered by applying ionic currents or withdrawal of the liquid, actively countering leakage of multiple channels would be prohibitively complex. Rather, blocking diffusion requires disrupting the aqueous phase with a phase barrier.

Actuation of a physical barrier is challenging in microfluidics. The classical Quake valve has been produced as small as 6 μm but requires multilayer soft lithography in monolithic structures (Araci and Quake, 2012). Solid barriers have been electrochemically opened but are not reversible (Chung et al., 2008). Air has been used to interrupt diffusion, but required a macroscopic gap which cannot be miniaturized or implanted (Zibek et al., 2010).

A further weakness of microfluidic stimulation is its low spatial resolution. Low spatial resolution hinders temporal resolution due to the so-called proximity effect (Iezzi and Finlayson, 2011). When diffusion is the primary transport mechanism, increasing distances cause exponential dilution and slowing of chemical signal transmission (e.g., latency of 1 s for 33 μm; Iezzi and Finlayson, 2011). Microfluidic or pipette-based release distant from target neurons clearly showed the latency of signal transmission (Peterman et al., 2004; Scott et al., 2013; Inayat et al., 2014). However, targeted iontophoretic stimulation of neuronal structures from ~100 nm pipette tips supports the possibility of fast chemical stimulation (Murnick et al., 2002).

No reported microfluidic technology can control single chemical release sites, and microfluidic structures provide insufficient resolution for communication with neurons. The challenge of controlling of chemical release sites for high resolution chemical release cannot be realized by microfluidics. Mimicry of physiological neurotransmission will require nanofluidic technology. The following section will discuss how nanopores could achieve synapse-like release.

## Nanofluidic chemical release

The emphasis on *nano*fluidics is not surprising upon reviewing the physical mechanisms of neurotransmission. Neurotransmission relies on vesicular release of neurotransmitters, orchestrated by complex systems of molecular biology in the presynaptic neuron (Purves et al., 2004). Vesicles, 50 nm in diameter, contain up to 40,000 neurotransmitter molecules (Van der Kloot, 1991). Vesicular release occurs at specialized regions of synapses called active zones, with diameters of 200–500 nm (Südhof, 2012). Action potentials drive release of vesicles as fast as 2000 Hz (Kaeser and Regehr, 2014); although not sustainable, this rate can provide an upper estimate of release rates. The product of these values suggests an upper approximation for neurotransmitter release at an active zone of ~10^−16^ mol/s. A helpful comparison: if each molecule is a monovalent ion, this represents a current of 10 pA. The dimensions of active zones and their central role in neurotransmission make them an intriguing target for mimicry with an artificial nanopore device.

Replacement of the presynaptic neuron by an artificial device will require the capability of sustained release of at least the rate achieved by active zones. Real connections between devices and biology are never ideal, so higher release rates may be required to overcome reduced proximity. Release rates calculated below predict that nanofluidic elements can achieve physiologically-relevant release rates.

A notable property of synaptic release is its absolute nature. Vesicles enclose neurotransmitter molecules, and chemical transport across the cell membrane is strictly regulated. This contrasts with the limited gating capabilities achieved in artificial devices. Progress in nanofluidics has focused on nanopores: channels narrower than 100 nm fabricated perpendicularly through thin membranes. Exploitation of the influence of surface properties can control transport of ions or fluid, although electrostatic or steric effects have not achieved absolute shut-off (Taghipoor et al., 2015). A suitable technique to prevent diffusion requires a barrier to interrupt the aqueous phase. Hydrophobic gating could provide such a mechanism. Reports of relevant phenomena are discussed below to provide a perspective on what may be possible.

### Nanofluidic release rates

**Figure 1:**
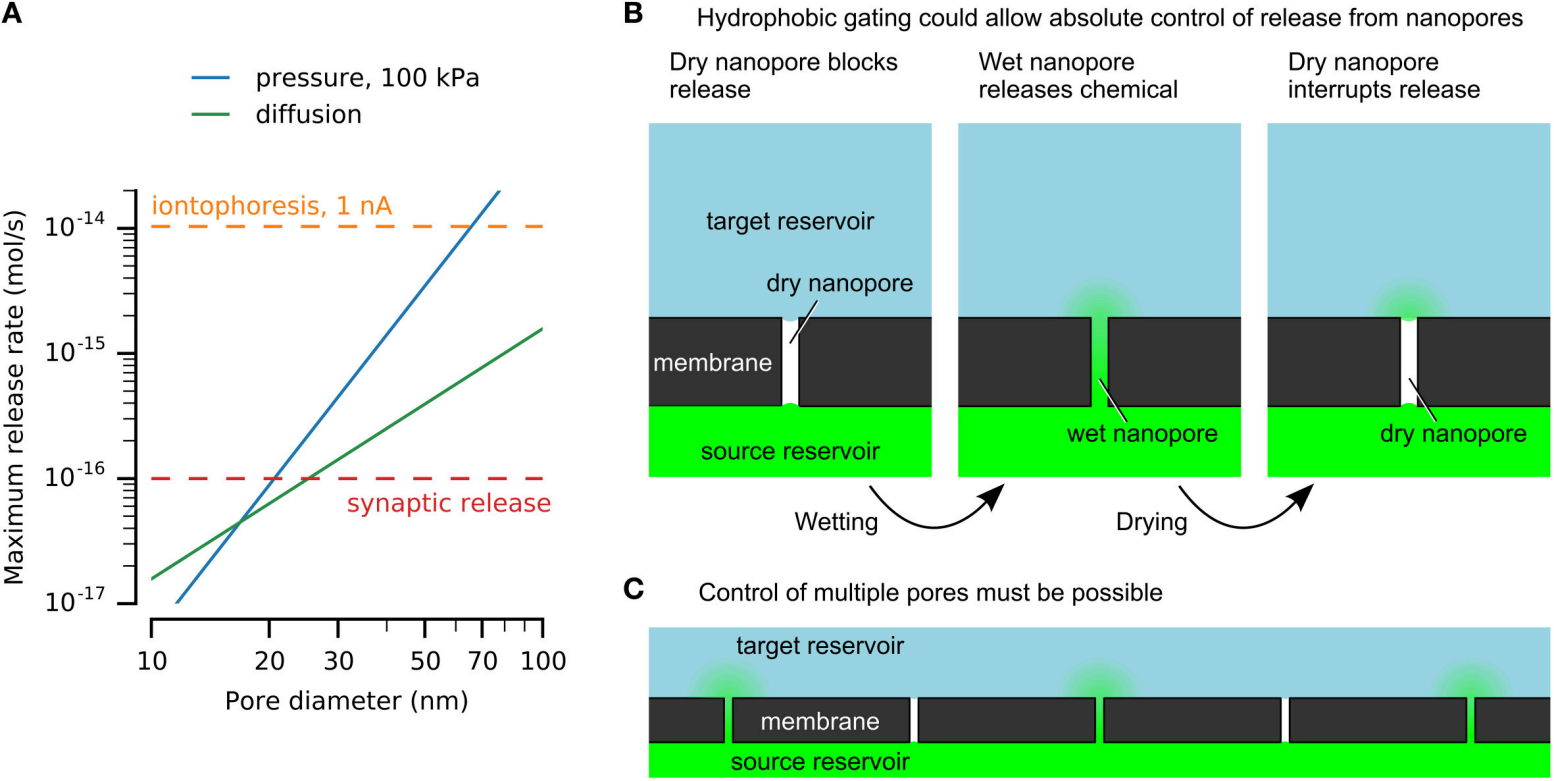
**Nanofluidics may enable chemical release similar to vesicular release at synaptic active zones. (A)** Nanopores can release physiologically-relevant quantities. Release rates by diffusion and pressure-driven flow through nanopores with varying diameters are compared here to an upper estimate for release from a single synaptic active zone and iontophoresis. Calculations used a pore length of 500 nm, source concentration of 100 mM, and diffusivity of 10^−^^9^ m^2^/s. **(B)** Hydrophobic gating of nanopores may enable absolute control of chemical release without leakage by diffusion. **(C)** Simultaneous control of large numbers of pores will be necessary for neuroprostheses.

An upper limit for vesicular release at single active zones was estimated above to be 10^−16^ mol/s, and provides a reference for discussion of nanofluidic release rates (Figure 1A). Another useful value is obtained from microiontophoresis, which can selectively stimulate single synapses to produce physiological responses (Murnick et al., 2002). Typical currents range from a few nanoamperes up to 100 nA (Lalley, 1999). Although direct quantification is challenging (Herr et al., 2008), converting 1 nA directly to monovalent ions is 10^−14^ mol/s (Figure 1A).

Diffusion and pressure may drive chemical release from a nanopore. Further discussion and derivation are available in the Supplementary Material. The release rate by diffusion, in mol/s, is

$$\begin{array}{l} n_{D}=\frac{\pi c_{0}Dd^{2}}{4L}, \end{array}$$

with source concentration *c*_0_, diffusivity *D*, and nanopore diameter *d* and length *L*. This expression assumes a linear concentration gradient along the length of the nanopore.

The release rate by applied pressure is

$$\begin{array}{l} n_{P}=\frac{\pi c_{0}\Delta Pd^{4}}{128\eta L}, \end{array}$$

with pressure Δ*P* and dynamic viscosity η. This expression assumes Hagen–Poiseuille flow without slip and neglects depletion or accumulation at the pore ends.

Example solutions are shown in Figure 1A for nanopores of various diameters, showing that nanopores can release chemicals at physiologically-relevant rates. This example uses a length of 500 nm, which would be sufficient to integrate electrodes or other functional elements. A 100 mM source is below synaptic vesicle concentrations. The release rates are sufficient to imitate synaptic transmission, given an appropriate method to turn release on and off.

### Nanofluidic gating

Hydrophobic gating achieves absolute disruption of chemical transport by control of a vapor-phase barrier (Figure 1B). Although ubiquitous in cell membrane protein pores (Aryal et al., 2015), achieving similar effects in artificial nanopores has proven more challenging. Spontaneous nucleation of bubbles is prohibited in pores larger than a few nanometers in diameter, regardless of length (Lefevre et al., 2004; Guillemot et al., 2012). Top-down nanofabrication cannot achieve sufficient precision to mimic these dimensions. For example, hydrophobic gating in protein pores is affected by sub-nanometer changes in dimensions or single amino acid residue substitutions (Yoshimura et al., 1999; Beckstein and Sansom, 2003; Birkner et al., 2012).

Reversible hydrophobic gating in wider artificial nanopores has been demonstrated in response to applied electric fields (Powell et al., 2011; Smirnov et al., 2011). The reversibility demonstrated in these larger pores relies on trapping of bubbles within the pores (Smirnov et al., 2010), and their removal makes wetting irreversible. Deliberate bubble trapping by constrictions or surface chemistry has been suggested but not yet demonstrated (Smirnov et al., 2010; Guillemot et al., 2012). A hydrophobic liquid could provide an alternative to vapor bubbles; this concept has been demonstrated in macroscopic nanoporous membranes (Hou et al., 2015) but not in single nanopores. Reversibility could be achieved by generating bubbles by plasmonic heating (Li et al., 2015), Joule heating (Nagashima et al., 2014), or electrolysis (Chen et al., 2015).

As nanofluidic effects arise from surface properties, precise nanometer-scale control of structure and surface will be necessary to achieve desired functions. A leading example of artificial nanopore fabrication is a wafer-scale process for sub-20-nm-diameter pores with integrated electrodes (Bai et al., 2014). While most applications use homogenous surface chemistry, for example by silane-based modification (Miles et al., 2013), the molecular topography of such surfaces must not be overlooked (Fadeev and McCarthy, 1999). Long-term applications will require sufficient stability of nanopores' structure and surface chemistry. Silicon nitride pores widen due to decomposition and dissolution (Rollings et al., 2015). In biological environments, protein adsorption presents further challenges (Yusko et al., 2011). Nanopores are usually investigated in isolation, and integration of arrays of nanopores remains a challenge. A recent report integrated five pores in individual microfluidic channels (Tahvildari et al., 2015).

These reports hint at what may be possible, while emphasizing the limited robustness and poor understanding of these effects. Robust and reversible nanopore electrowetting has not been demonstrated, and the mechanisms of electric-field-induced wetting of nanopores are not known. However, a generic mechanism can be envisioned (Figure 1B). A hydrophobic barrier will block chemical transport by formation of a phase barrier of vapor or a hydrophobic liquid. The Young–Laplace law explains the resistance to wetting of hydrophobic nanopores (Lee and Karnik, 2010), and has been verified in pores as narrow as 2.6 nm (Lefevre et al., 2004). A stimulus will form a water channel across the hydrophobic barrier, allowing chemical transport. This may be achieved by modulating the pore's surface energy or by applying sufficient pressure or voltage to force water into the pore. Dewetting may occur spontaneously or may be driven, for example by heating. For applications in neuroprotheses, control of many nanopores simultaneously must be achieved (Figure 1C).

## Propagation of chemical signals

Propagation of chemical signals occurs throughout the nervous system with diverse spatial and temporal scales (Syková and Nicholson, 2008; Vizi and Lendvai, 2008; Rusakov et al., 2011). Neurotransmitters diffuse across the synaptic cleft (~20 nm) faster than 1 μs; diffusion also drives volume transmission over larger distances at time scales of minutes or longer. Extracellular diffusion can be studied by iontophoretic injection and electrochemical detection of a tracer molecule (Nicholson et al., 1979). The diffusion equation can be modified to consider extracellular volume fraction and tortuosity, but such approximations are invalid in specific micro- or nanoscale geometries. Moreover, uptake and enzymatic reactions influence extracellular chemical signals.

The synapses of cone photoreceptor cells demonstrate the potential complexity of nanoscale chemical signal transmission (Regus-Leidig and Brandstätter, 2012). Neurotransmitter release from these neurons addresses multiple postsynaptic cells, whose responses depend on their spatial proximity to the active zone. Postsynaptic dendrites localized at the active zone receive rapid high neurotransmitter concentrations, while cells which contact the photoreceptors farther from the active zone receive smoother, lower concentrations. The responses of these postsynaptic neurons correlate with the nanoscale propagation of neurotransmitter.

The proximity effect is a challenge for chemical stimulation: diffusive transport to larger distances requires exponentially longer times and leads to exponential dilution (Iezzi and Finlayson, 2011). The dimensions of microfluidic chemical stimulation devices suggest a limit of seconds to minutes. However, the chemical communication mechanisms of the brain prove the capability of delivering chemical information over diverse spatial and temporal dimensions. Slow volume transmission across large distances (Syková and Nicholson, 2008) could provide a target for neuromodulatory chemical neuroprostheses. Fast, high resolution stimulation requires intimate contact with cells (Murnick et al., 2002).

**Figure 2:**
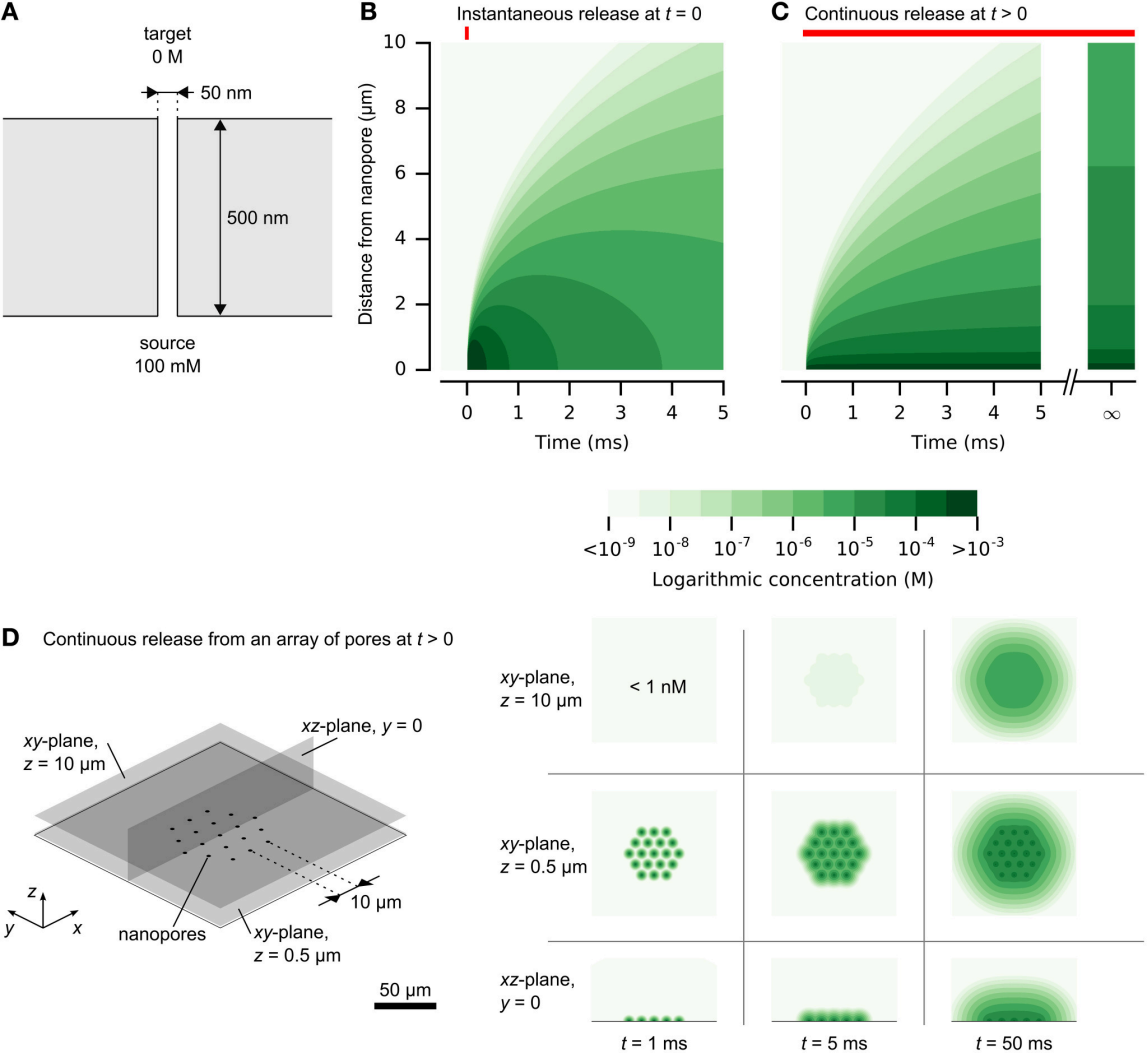
**Release and spread of chemical signals from nanopores by diffusion. (A)** The physical setup of a single nanopore with diameter of 50 nm and length of 500 nm. **(B)** Propagation of an instantaneous chemical impulse of 10^6^ molecules at *t* = 0. Concentrations 1 μm away from the nanopore source rise and fall by orders of magnitudes within milliseconds**. (C)** Propagation of constant release from a nanopore, illustrated in **(A)**, turned on at *t* > 0. A high concentration is established quickly near the nanopore. At larger distances, the concentration approaches a steady state, which is diluted by orders of magnitude in comparison to the concentration within 1 μm of the nanopore. **(D)** A dense array of nanopores can be resolved at close distances. Here, the release rate at each nanopore is the same as in **(C)** and the nanopores are separated by 10 μm. Color scale is logarithmic and shown in discrete steps for clarity. A reproduction with a linear color scale is available as Figure 1S in the Supplementary Material.

Chemical signal propagation from nanopores may be illustrated by analytical solutions to the diffusion equation (Crank, 1975). Expressions for instantaneous or continuous release from a point source are described in the Supplementary Material and illustrated in Figure 2. Figure 2B shows the rapid rise and fall of a chemical impulse, with micrometer-scale resolution and millisecond-scale time course. Figure 2C shows the propagation of constant diffusion from a nanopore with diameter of 50 nm and length of 500 nm, and release by diffusion only (4·10^−16^ mol/s). High concentrations rapidly establish near the source, while spread to larger distances is slower. Concentrations at larger distances are limited by continuous diffusion to a steady-state limit. Long release times cannot produce concentrated signals at large distances from a nanopore.

The analytical solution for continuous release also provides an estimate of potential density of independent nanopores. Figure 2D illustrates this for an array of nanopores with a pitch of 10 μm. At close distances, the signals are clearly resolved, while larger distances obscure the individual signals. This analytical solution provides only an estimate, without considering interference of the neighboring pores. Improved accuracy of time-varying chemical release from multiple nanopores could be obtained by numerical simulation, which could permit inclusion of cellular structures (Hepburn et al., 2012).

## Conclusion/outlook

The clinical success of electrical neuroprostheses has proven the possibility to treat neurological disorders with artificial devices, while also revealing limitations of addressing complex physiology with comparatively simple electrical methods. Neurotransmitter-based stimulation could enable the ultimate neuroprosthesis by addressing neurons with their own chemical language.

The challenges to be overcome must not be underestimated. The field of microfluidics remains immature in comparison to microelectronics (Becker, 2009; Whitesides, 2011). Nanofluidics has remained especially unexplored due to limitations of fabrication (Whitesides, 2011). Neurotransmitter-based stimulation will require continued developments of microfluidics and nanofluidics. The development of nanoelectrodes (Angle et al., 2015) may provide an indication of the challenges involved in interfacing nanoscale devices with biological systems. However, while electrode development may benefit from expertise of the nanoelectronics industry, similarly mature expertise in nanofluidics does not exist.

A robust nanofluidic gating mechanism is required. Hydrophobic gating may provide the mechanism, although a robust realization of this effect may be different from what has been reported until now. Reliable fabrication and operation of thousands of individual pores must be demonstrated. Large scale integration of individually controlled nanopores with microfluidic and electrical control will be necessary. Certainly, many generations of technology development will precede the realization of the goal of chemical neuroprostheses.

Some challenges can be predicted. Long-term operation in aggressive biological environments will be necessary for the goal of neuroprostheses. The requirement for intimate proximity with target neurons may require advanced biochemical functionalization of the device or release of neurotrophic factors to encourage the neurons' acceptance of an artificial device. Removal of chemical signals must be investigated to avoid excitotoxicity, although surrounding cells may accomplish this, for example by the widely-expressed excitatory amino acid transporters in the retina (Iezzi and Finlayson, 2011).

The complexity of the brain raises an important question: Do we understand the brain well-enough to rationally stimulate it with high resolution chemical signals? Stimulation of well-understood structures including the retina or sensory cortex should be possible (Iezzi and Finlayson, 2011; Bensmaia, 2015). However, deep brain stimulation protocols continue to rely on trial-and-error optimization with patient feedback (Kringelbach et al., 2007). Neurons integrate inputs of up to hundreds of thousands of synapses (Stuart and Spruston, 2015). If nanopores are envisioned to replace single synaptic inputs, an artificial device cannot be expected to interface with complex brain areas. However, as “megascience” efforts turn their focus to neuroscience, coming years may see an acceleration of our understanding of the brain (Grillner, 2014) which may reveal new possibilities for treatment of neurological disorders by artificial chemical stimulation.

## Author contributions

PJ and MS developed the concepts described in this work. PJ wrote the manuscript. PJ and MS revised the work and approved the final version.

### Conflict of interest statement

The authors declare that the research was conducted in the absence of any commercial or financial relationships that could be construed as a potential conflict of interest.

## References

[B1] Adekunle A. N. Adkins A. Wang W. Kaplan H. J. Fernandez J. Castro D. (2015). Integration of perforated subretinal prostheses with retinal tissue. Transl. Vis. Sci. Technol. 4, 1–10. 10.1167/tvst.4.4.5 PMC4539203 26290776

[B2] Altuna A. Bellistri E. Cid E. Aivar P. Gal B. Berganzo J.. (2013). SU-8 based microprobes for simultaneous neural depth recording and drug delivery in the brain. Lab Chip 13, 1422–1430. 10.1039/c3lc41364k 23407672

[B3] Angle M. R. Cui B. Melosh N. A. (2015). Nanotechnology and neurophysiology. Curr. Opin. Neurobiol. 32, 132–140. 10.1016/j.conb.2015.03.014 25889532

[B4] Araci I. E. Quake S. R. (2012). Microfluidic very large scale integration (mVLSI) with integrated micromechanical valves. Lab Chip 12, 2803. 10.1039/c2lc40258k 22714259

[B5] Aryal P. Sansom M. S. P. Tucker S. J. (2015). Hydrophobic gating in ion channels. J. Mol. Biol. 427, 121–130. 10.1016/j.jmb.2014.07.030 25106689 PMC4817205

[B6] Bai J. Wang D. Nam S. Peng H. Bruce R. Gignac L.. (2014). Fabrication of sub-20 nm nanopore arrays in membranes with embedded metal electrodes at wafer scales. Nanoscale 6, 8900–8906. 10.1039/C3NR06723H 24964839

[B7] Barrett J. M. Berlinguer-Palmini R. Degenaar P. (2014). Optogenetic approaches to retinal prosthesis. Vis. Neurosci. 31, 345–354. 10.1017/S0952523814000212 25100257 PMC4161214

[B8] Becker H. (2009). Hype, hope and hubris: the quest for the killer application in microfluidics. Lab Chip 9, 2119–2122. 10.1039/b911553f 19606286

[B9] Beckstein O. Sansom M. S. P. (2003). Liquid-vapor oscillations of water in hydrophobic nanopores. Proc. Natl. Acad. Sci. U.S.A. 100, 7063–7068. 10.1073/pnas.1136844100 12740433 PMC165830

[B10] Bensmaia S. J. (2015). Biological and bionic hands: natural neural coding and artificial perception. Philos. Trans. R. Soc. B Biol. Sci. 370, 20140209 10.1098/rstb.2014.0209 PMC4528821 26240424

[B11] Birkner J. P. Poolman B. Koçer A. (2012). Hydrophobic gating of mechanosensitive channel of large conductance evidenced by single-subunit resolution. Proc. Natl. Acad. Sci. U.S.A. 109, 12944–12949. 10.1073/pnas.1205270109 22826215 PMC3420157

[B12] Borton D. Micera S. Millán J. D. R. Courtine G. (2013). Personalized neuroprosthetics. Sci. Transl. Med. 5, 210rv2. 10.1126/scitranslmed.3005968 24197737

[B79] Bourzac K. (2016). Texas Woman is the First Person to Undergo Optogenetic Therapy. MIT Technology Review. Available online at: https://www.technologyreview.com/s/601067/texas-woman-is-the-first-person-to-undergo-optogenetic-therapy/

[B13] Chen Q. Luo L. White H. S. (2015). Electrochemical generation of a hydrogen bubble at a recessed platinum nanopore electrode. Langmuir 31, 4573–4581. 10.1021/acs.langmuir.5b00234 25811080

[B14] Chung A. J. Kim D. Erickson D. (2008). Electrokinetic microfluidic devices for rapid, low power drug delivery in autonomous microsystems. Lab Chip 8, 330–338. 10.1039/B713325A 18231674

[B15] Crank J. (1975). The Mathematics of Diffusion, 2nd Edn. London: Oxford University Press.

[B16] Durand D. M. (2000). Electric stimulation of excitable tissue, in The Biomedical Engineering Handbook, ed Bronzino J. D. (Boca Raton, FL: CRC Press), 1–22.

[B17] Eiber C. D. Lovell N. H. Suaning G. J. (2013). Attaining higher resolution visual prosthetics: a review of the factors and limitations. J. Neural Eng. 10:011002. 10.1088/1741-2560/10/1/011002 23337266

[B18] Fadeev A. Y. McCarthy T. J. (1999). Trialkylsilane monolayers covalently attached to silicon surfaces: wettability studies indicating that molecular topography contributes to contact angle hysteresis. Langmuir 15, 3759–3766. 10.1021/la981486o

[B19] Featherstone D. E. (2010). Intercellular glutamate signaling in the nervous system and beyond. ACS Chem. Neurosci. 1, 4–12. 10.1021/cn900006n 22778802 PMC3368625

[B20] Finlayson P. G. Iezzi R. (2010). Glutamate stimulation of retinal ganglion cells in normal and s334ter-4 rat retinas: a candidate for a neurotransmitter-based retinal prosthesis. Invest. Ophthalmol. Vis. Sci. 51, 3619–3628. 10.1167/iovs.09-4877 20164453 PMC2904014

[B21] Frey O. Holtzman T. McNamara R. M. Theobald D. E. H. van der Wal P. D. de Rooij N. F. (2011). Simultaneous neurochemical stimulation and recording using an assembly of biosensor silicon microprobes and SU-8 microinjectors. Sensors Actuators B Chem. 154, 96–105. 10.1016/j.snb.2010.01.034

[B22] Gerwig R. Fuchsberger K. Schroeppel B. Link G. S. Heusel G. Kraushaar U. (2012). PEDOT-CNT composite microelectrodes for recording and electrostimulation applications: fabrication, morphology, and electrical properties. Front. Neuroeng. 5:8 10.3389/fneng.2012.00008 PMC3343311 22586394

[B23] Grillner S. (2014). Megascience efforts and the brain. Neuron 82, 1209–1211. 10.1016/j.neuron.2014.05.045 24945766

[B24] Guillemot L. Biben T. Galarneau A. Vigier G. Charlaix É. (2012). Activated drying in hydrophobic nanopores and the line tension of water. Proc. Natl. Acad. Sci. U.S.A. 109, 19557–19562. 10.1073/pnas.1207658109 23144219 PMC3511739

[B25] Hai A. Shappir J. Spira M. E. (2010). Long-term, multisite, parallel, in-cell recording and stimulation by an array of extracellular microelectrodes. J. Neurophysiol. 104, 559–568. 10.1152/jn.00265.2010 20427620

[B26] Hepburn I. Chen W. Wils S. De Schutter E. (2012). STEPS: efficient simulation of stochastic reaction-diffusion models in realistic morphologies. BMC Syst. Biol. 6:36 10.1186/1752-0509-6-36 22574658 PMC3472240

[B27] Herman M. A. Jahr C. E. (2007). Extracellular glutamate concentration in hippocampal slice. J. Neurosci. 27, 9736–9741. 10.1523/JNEUROSCI.3009-07.2007 17804634 PMC2670936

[B28] Herr N. R. Kile B. M. Carelli R. M. Wightman R. M. (2008). Electroosmotic flow and its contribution to iontophoretic delivery. Anal. Chem. 80, 8635–8641. 10.1021/ac801547a 18947198 PMC2772194

[B29] Hirsch J. Curcio C. A. (1989). The spatial resolution capacity of human foveal retina. Vision Res. 29, 1095–1101. 10.1016/0042-6989(89)90058-8 2617858

[B30] Hou X. Hu Y. Grinthal A. Khan M. Aizenberg J. (2015). Liquid-based gating mechanism with tunable multiphase selectivity and antifouling behaviour. Nature 519, 70–73. 10.1038/nature14253 25739629

[B31] Hunter J. D. (2007). Matplotlib: a 2D graphics environment. Comput. Sci. Eng. 9, 99–104. 10.1109/MCSE.2007.55

[B32] Iezzi R. Finlayson P. G. (2011). Neurotransmitter stimulation for retinal prosthesis: the artificial synapse chip, in Visual Prosthetics, ed Dagnelie G. (Boston, MA: Springer), 173–191. 10.1007/978-1-4419-0754-7_9

[B33] Inayat S. Rountree C. M. Troy J. B. Saggere L. (2014). Chemical stimulation of rat retinal neurons: feasibility of an epiretinal neurotransmitter-based prosthesis. J. Neural Eng. 12:016010. 10.1088/1741-2560/12/1/016010 25504758

[B34] Kaeser P. S. Regehr W. G. (2014). Molecular mechanisms for synchronous, asynchronous, and spontaneous neurotransmitter release. Annu. Rev. Physiol. 76, 333–363. 10.1146/annurev-physiol-021113-170338 24274737 PMC4503208

[B35] Kringelbach M. L. Jenkinson N. Owen S. L. F. Aziz T. Z. (2007). Translational principles of deep brain stimulation. Nat. Rev. Neurosci. 8, 623–635. 10.1038/nrn2196 17637800

[B36] Lalley P. M. (1999). Microiontophoresis and pressure ejection, in Modern Techniques in Neuroscience Research, ed Pitman R. M. (Berlin; Heidelberg: Springer), 193–212. 10.1007/978-3-642-58552-4_7

[B37] Lee J. Karnik R. (2010). Desalination of water by vapor-phase transport through hydrophobic nanopores. J. Appl. Phys. 108, 044315 10.1063/1.3419751

[B38] Lefevre B. Saugey A. Barrat J. L. Bocquet L. Charlaix E. Gobin P. F.. (2004). Intrusion and extrusion of water in hydrophobic mesopores. J. Chem. Phys. 120, 4927–4938. 10.1063/1.1643728 15267355

[B39] Li Y. Nicoli F. Chen C. Lagae L. Groeseneken G. Stakenborg T.. (2015). Photoresistance switching of plasmonic nanopores. Nano Lett. 15, 776–782. 10.1021/nl504516d 25514824 PMC4296925

[B40] Lorach H. Goetz G. Smith R. Lei X. Mandel Y. Kamins T.. (2015). Photovoltaic restoration of sight with high visual acuity. Nat. Med. 21, 476–482. 10.1038/nm.3851 25915832 PMC4601644

[B41] Lozano A. M. Lipsman N. (2013). Probing and regulating dysfunctional circuits using deep brain stimulation. Neuron 77, 406–424. 10.1016/j.neuron.2013.01.020 23395370

[B42] Merrill D. R. Bikson M. Jefferys J. G. R. (2005). Electrical stimulation of excitable tissue: design of efficacious and safe protocols. J. Neurosci. Methods 141, 171–198. 10.1016/j.jneumeth.2004.10.020 15661300

[B43] Miles B. N. Ivanov A. P. Wilson K. A. Doǧan F. Japrung D. Edel J. B. (2013). Single molecule sensing with solid-state nanopores: novel materials, methods, and applications. Chem. Soc. Rev. 42, 15–28. 10.1039/C2CS35286A 22990878

[B44] Murnick J. G. Dubé G. Krupa B. Liu G. (2002). High-resolution iontophoresis for single-synapse stimulation. J. Neurosci. Methods 116, 65–75. 10.1016/S0165-0270(02)00028-6 12007984

[B45] Nagashima G. Levine E. V. Hoogerheide D. P. Burns M. M. Golovchenko J. A. (2014). Superheating and homogeneous single bubble nucleation in a solid-state nanopore. Phys. Rev. Lett. 113, 024506. 10.1103/PhysRevLett.113.024506 25062192 PMC4113017

[B46] Nicholson C. Phillips J. M. Gardner-Medwin A. R. (1979). Diffusion from an iontophoretic point source in the brain: role of tortuosity and volume fraction. Brain Res. 169, 580–584. 10.1016/0006-8993(79)90408-6 445169

[B47] Penn R. D. Kroin J. S. (1985). Continuous intrathecal baclofen for severe spasticity. Lancet 2, 125–127. 10.1016/S0140-6736(85)90228-4 2862320

[B48] Peterman M. C. Noolandi J. Blumenkranz M. S. Fishman H. A. (2004). Localized chemical release from an artificial synapse chip. Proc. Natl. Acad. Sci. U.S.A. 101, 9951–9954. 10.1073/pnas.0402089101 15218102 PMC454196

[B49] Pongrácz A. Fekete Z. Márton G. Bérces Z. Ulbert I. Fürjes P. (2013). Deep-brain silicon multielectrodes for simultaneous *in vivo* neural recording and drug delivery. Sensors Actuators B Chem. 189, 97–105. 10.1016/j.snb.2013.01.032

[B50] Powell M. R. Cleary L. Davenport M. Shea K. J. Siwy Z. S. (2011). Electric-field-induced wetting and dewetting in single hydrophobic nanopores. Nat. Nanotechnol. 6, 798–802. 10.1038/nnano.2011.189 22036811

[B51] Purves D. Augustine G. J. Fitzpatrick D. Hall W. C. LaMantia A.-S. McNamara J. O. (eds.). (2004). Neuroscience, 3rd Edn. Sunderland, MA: Sinauer Associates Available online at: http://www.ncbi.nlm.nih.gov/books/NBK10799/ (Accessed April 25, 2015).

[B52] Qing Q. Jiang Z. Xu L. Gao R. Mai L. Lieber C. M. (2013). Free-standing kinked nanowire transistor probes for targeted intracellular recording in three dimensions. Nat. Nanotechnol. 9, 142–147. 10.1038/nnano.2013.273 24336402 PMC3946362

[B53] Regus-Leidig H. Brandstätter J. H. (2012). Structure and function of a complex sensory synapse. Acta Physiol. (Oxf.) 204, 479–486. 10.1111/j.1748-1716.2011.02355.x 21880116

[B80] RetroSense Therapeutics (2015). RST-001 Phase I/II Trial for Retinitis Pigmentosa. Available online at: https://clinicaltrials.gov/ct2/show/study/NCT02556736

[B54] Rollings R. Graef E. Walsh N. Nandivada S. Benamara M. Li J. (2015). The effects of geometry and stability of solid-state nanopores on detecting single DNA molecules. Nanotechnology 26:044001. 10.1088/0957-4484/26/4/044001 25556317 PMC4288979

[B55] Rusakov D. A. Savtchenko L. P. Zheng K. Henley J. M. (2011). Shaping the synaptic signal: molecular mobility inside and outside the cleft. Trends Neurosci. 34, 359–369. 10.1016/j.tins.2011.03.002 21470699 PMC3133640

[B56] Samba R. Herrmann T. Zeck G. (2015). PEDOT-CNT coated electrodes stimulate retinal neurons at low voltage amplitudes and low charge densities. J. Neural Eng. 12, 016014. 10.1088/1741-2560/12/1/016014 25588201

[B57] Sanchez J. (2015). Neurotechnology, in Wait, What? (St. Louis, MI: DARPA). Available online at: http://archive.darpa.mil/WaitWhat/

[B58] Scimemi A. Beato M. (2009). Determining the neurotransmitter concentration profile at active synapses. Mol. Neurobiol. 40, 289–306. 10.1007/s12035-009-8087-7 19844813 PMC2777263

[B59] Scott A. Au A. K. Vinckenbosch E. Folch A. (2013). A microfluidic D-subminiature connector. Lab Chip 13, 2036–2039. 10.1039/c3lc50201e 23584282 PMC3786702

[B60] Shannon R. V. (2012). Advances in auditory prostheses. Curr. Opin. Neurol. 25, 61–66. 10.1097/WCO.0b013e32834ef878 22157109 PMC4123811

[B61] Smirnov S. N. Vlassiouk I. V. Lavrik N. V. (2011). Voltage-gated hydrophobic nanopores. ACS Nano 5, 7453–7461. 10.1021/nn202392d 21838311

[B62] Smirnov S. Vlassiouk I. Takmakov P. Rios F. (2010). Water confinement in hydrophobic nanopores. Pressure-induced wetting and drying. ACS Nano 4, 5069–5075. 10.1021/nn101080k 20690599

[B63] Stett A. Mai A. Herrmann T. (2007). Retinal charge sensitivity and spatial discrimination obtainable by subretinal implants: key lessons learned from isolated chicken retina. J. Neural Eng. 4, S7–S16. 10.1088/1741-2560/4/1/s02 17325418

[B64] Stingl K. Bartz-Schmidt K. U. Besch D. Braun A. Bruckmann A. Gekeler F.. (2013). Artificial vision with wirelessly powered subretinal electronic implant alpha-IMS. Proc. R. Soc. B Biol. Sci. 280, 20130077. 10.1098/rspb.2013.0077 23427175 PMC3619489

[B65] Stuart G. J. Spruston N. (2015). Dendritic integration: 60 years of progress. Nat. Neurosci. 18, 1713–1721. 10.1038/nn.4157 26605882

[B66] Südhof T. C. (2012). The presynaptic active zone. Neuron 75, 11–25. 10.1016/j.neuron.2012.06.012 22794257 PMC3743085

[B67] Sun W. Shchepakin D. Kalachev L. V. Kavanaugh M. P. (2014). Glutamate transporter control of ambient glutamate levels. Neurochem. Int. 73, 146–151. 10.1016/j.neuint.2014.04.007 24768447

[B68] Syková E. Nicholson C. (2008). Diffusion in brain extracellular space. Physiol. Rev. 88, 1277–1340. 10.1152/physrev.00027.2007 18923183 PMC2785730

[B69] Taghipoor M. Bertsch A. Renaud P. (2015). Thermal control of ionic transport and fluid flow in nanofluidic channels. Nanoscale 7, 18799–18804. 10.1039/C5NR05409E 26507947

[B70] Tahvildari R. Beamish E. Tabard-Cossa V. Godin M. (2015). Integrating nanopore sensors within microfluidic channel arrays using controlled breakdown. Lab Chip 15, 1407–1411. 10.1039/C4LC01366B 25631885

[B71] Van der Kloot W. (1991). The regulation of quantal size. Prog. Neurobiol. 36, 93–130. 10.1016/0301-0082(91)90019-W 1847748

[B72] Vizi E. S. Lendvai B. (2008). Synaptic and nonsynaptic release of transmitters, in Handbook of Neurochemistry and Molecular Neurobiology: Neurotransmitter Systems, eds Lajtha N. S. A. Vizi S. E. (Springer). Available online at: http://www.springer.com/gp/book/9780387303512

[B73] Whitesides G. M. (2011). What comes next? Lab Chip 11, 191–193. 10.1039/C0LC90101F 21152593

[B74] Yoshimura K. Batiza A. Schroeder M. Blount P. Kung C. (1999). Hydrophilicity of a single residue within MscL correlates with increased channel mechanosensitivity. Biophys. J. 77, 1960–1972. 10.1016/S0006-3495(99)77037-2 10512816 PMC1300477

[B75] Yusko E. C. Johnson J. M. Majd S. Prangkio P. Rollings R. C. Li J.. (2011). Controlling protein translocation through nanopores with bio-inspired fluid walls. Nat. Nanotechnol. 6, 253–260. 10.1038/nnano.2011.12 21336266 PMC3071889

[B76] Zerangue N. Kavanaugh M. P. (1996). Flux coupling in a neuronal glutamate transporter. Nature 383, 634–637. 10.1038/383634a0 8857541

[B77] Zibek S. Hagmeyer B. Stett A. Stelzle M. (2010). Chemical stimulation of adherent cells by localized application of acetylcholine from a microfluidic system. Front. Neuroeng. 3:113. 10.3389/fneng.2010.00113 21151808 PMC2999818

[B78] Zrenner E. (2013). Fighting blindness with microelectronics. Sci. Transl. Med. 5, 210ps16. 10.1126/scitranslmed.3007399 24197733

