# Corrigendum: Can Nanofluidic Chemical Release Enable Fast, High Resolution Neurotransmitter-Based Neurostimulation?

**DOI:** 10.3389/fnins.2016.00341

**Published:** 2016-07-21

**Authors:** Peter D. Jones, Martin Stelzle

**Affiliations:** BioMEMS & Sensors, Natural and Medical Sciences Institute, University of TübingenReutlingen, Germany

**Keywords:** nanofluidic, nanopore, microfluidic, neurotransmitter, neurotransmission, chemical neuroprosthesis, hydrophobic gating, artificial synapse

We cited a publication by Scott et al. which was not the correct reference. The correct reference is “A microfluidic microelectrode array for simultaneous electrophysiology, chemical stimulation, and imaging of brain slices” (Scott et al., 2013). We apologize for the mistake.

## Author contributions

All authors listed, have made substantial, direct and intellectual contribution to the work, and approved it for publication.

### Conflict of interest statement

The authors declare that the research was conducted in the absence of any commercial or financial relationships that could be construed as a potential conflict of interest.
