# Supplementary material for: Can Nanofluidic Chemical Release Enable Fast, High Resolution Neurotransmitter-Based Neurostimulation?
Source: Front Neurosci. 2016 Mar 31;10:138. doi: 10.3389/fnins.2016.00138 (PMC4815362; doi:10.3389/fnins.2016.00138)
Supplement: Supplementary file 1 [file DataSheet1.PDF]

Supplementary Material

Can nanofluidic chemical release enable fast, high resolution neurotransmitter-based neurostimulation?

Peter D. Jones*, Martin Stelzle

NMI Natural and Medical Sciences Institute at the University of Tübingen, Reutlingen, Germany

*** Correspondence:** Peter D. Jones: peter.jones@nmi.de

1 Release from a nanopore

The molecular release from a nanopore will follow (Babakinejad et al., 2013)

$$\boldsymbol{J} = -D\nabla c + \boldsymbol{u}c_0 \tag{1.1}$$

with flux $\boldsymbol{J}$, diffusivity D, concentration c, reservoir concentration c_0, and fluid velocity $\boldsymbol{u}$. Pressure-driven flow will determine $\boldsymbol{u}$. This discussion will neglect electrokinetic effects, which are more challenging to quantify (Herr et al., 2008).

For a straightforward analytical solution, diffusion and pressure-driven must be considering individually. Release by diffusion is driven by the gradient of concentration. By considering the nanopore as a one-dimensional system with a negligible gradient at its ends, the concentration will drop linearly along its axis, so

$$\nabla c = -\frac{c_0}{L}, \tag{1.2}$$

with pore length L.

If flow is driven through the pore by a pressure differential, the assumptions supporting this linear concentration gradient will be invalid. Therefore, individual solutions for diffusion and pressure-driven release will be discussed. These solutions cannot be summed to determine the combined release due to pressure and diffusion.

From (1.1), the diffusion contribution is

$$\boldsymbol{J} = -D\nabla c. \tag{1.3}$$

Multiplying by the cross-sectional area of the pore and substituting the concentration gradient from (1.2) gives an expression for the release rate by diffusion,

$$n_D = \frac{\pi c_0 D d^2}{4L} \quad (1.4)$$

with pore diameter *d*.

From (1.1), the pressure-driven contribution is

$$\boldsymbol{J} = \boldsymbol{u} c_0. \quad (1.5)$$

The fluid velocity in a cylindrical channel due to pressure-driven flow can be integrated as the volumetric flow rate *Q*, so that

$$n_P = Q c_0. \quad (1.6)$$

According to the Hagen–Poiseuille law (Tritton, 1988),

$$Q = \frac{\pi d^4 \Delta P}{128 \eta L} \quad (1.7)$$

with pressure ΔP and dynamic viscosity η. For small hydrophobic pores, higher flow rates can result from slip of the fluid along the walls (Cottin-Bizonne et al., 2005), but this will be neglected here. The pressure-driven release rate in mol/s is

$$n_P = \frac{\pi c_0 d^4 \Delta P}{128 \eta L}. \quad (1.8)$$

2 Propagation of chemical signals

The expressions here apply to release from a nanopore in a planar substrate, and are doubled as diffusion into the substrate is prohibited. For release of an instantaneous chemical impulse of a quantity *N* from a point source at $t = 0$, the concentration at any distance *r* and time $t > 0$ can be calculated according to (Crank, 1975)

$$c(r,t) = \frac{N}{4(\pi D t)^{3/2}} \exp\left(\frac{-r^2}{4Dt}\right). \quad (2.1)$$

An example is illustrated in Figure 2(B) for 10^6 molecules released at $t = 0$.

Another useful solution (Crank, 1975) is for constant diffusion from a point source releasing at a constant rate of *n* in mol/s at $t > 0$

$$c(r,t) = \frac{n}{2\pi Dr}\operatorname{erfc}\left(\frac{r}{2\sqrt{Dt}}\right), \tag{2.2}$$

with the complementary error function erfc. Figure 2(C) illustrates the propagation of such a signal. High concentrations are rapidly established near the source, while spread to larger distances is slower. Furthermore, concentrations at larger distances are limited by continuous diffusion, which sets a steady-state limit of

$$\lim_{t\to\infty} c(r,t) = \frac{n}{2\pi Dr}. \tag{2.3}$$

Continuous spreading of the concentrated chemical signal near a nanopore must remain within this limit, and long release times will not result in concentrated signals at large distances (Figure 2(C), $t \to \infty$).

Equation (2.2) can also provide an estimate of potential density of independent nanopores. Figure 2(D) illustrates this by solving equation (2.2) for an array of nanopores separated by 10 μm. At close distances, the signals are clearly resolved, while larger distances obscure the individual signals.

In biological environments, diffusion is affected by the tortuosity and the reduced volume fraction of the extracellular space. Although specific nanometre- or micrometre-scale features must be considered for exact solutions, these properties can be approximated at larger scales (Syková and Nicholson, 2008). Pressure-driven release would include advection, which is not considered in this analysis and can be neglected at sufficient distance from the point source for low flow rates (Babakinejad et al., 2013). Improved accuracy of time-varying chemical release from multiple nanopores could be obtained by numerical simulation, which could permit inclusion of cellular structures (Hepburn et al., 2012).

3 Release and spread of chemical signals from nanopores by diffusion, linear scale

Figure 1S reproduces Figure 2 with a linear color scale, rather than a logarithmic scale. The logarithmic scale provides more information, while the linear scale provides a more intuitive view of the localized concentrations expected from nanopores. All other details, including axes limits, are unchanged. The temporal localization in Figure 1S(B) and the spatial localization in Figure 1S(B) and (C) are more clear with a linear scale. The dimensions and axes limits in Figure 2(D) were chosen to provide information with a logarithmic scale. The corresponding images in Figure 1S(D) provide only limited information, with concentrated points visible in the *xy*-plane at $z = 0.5$ μm.

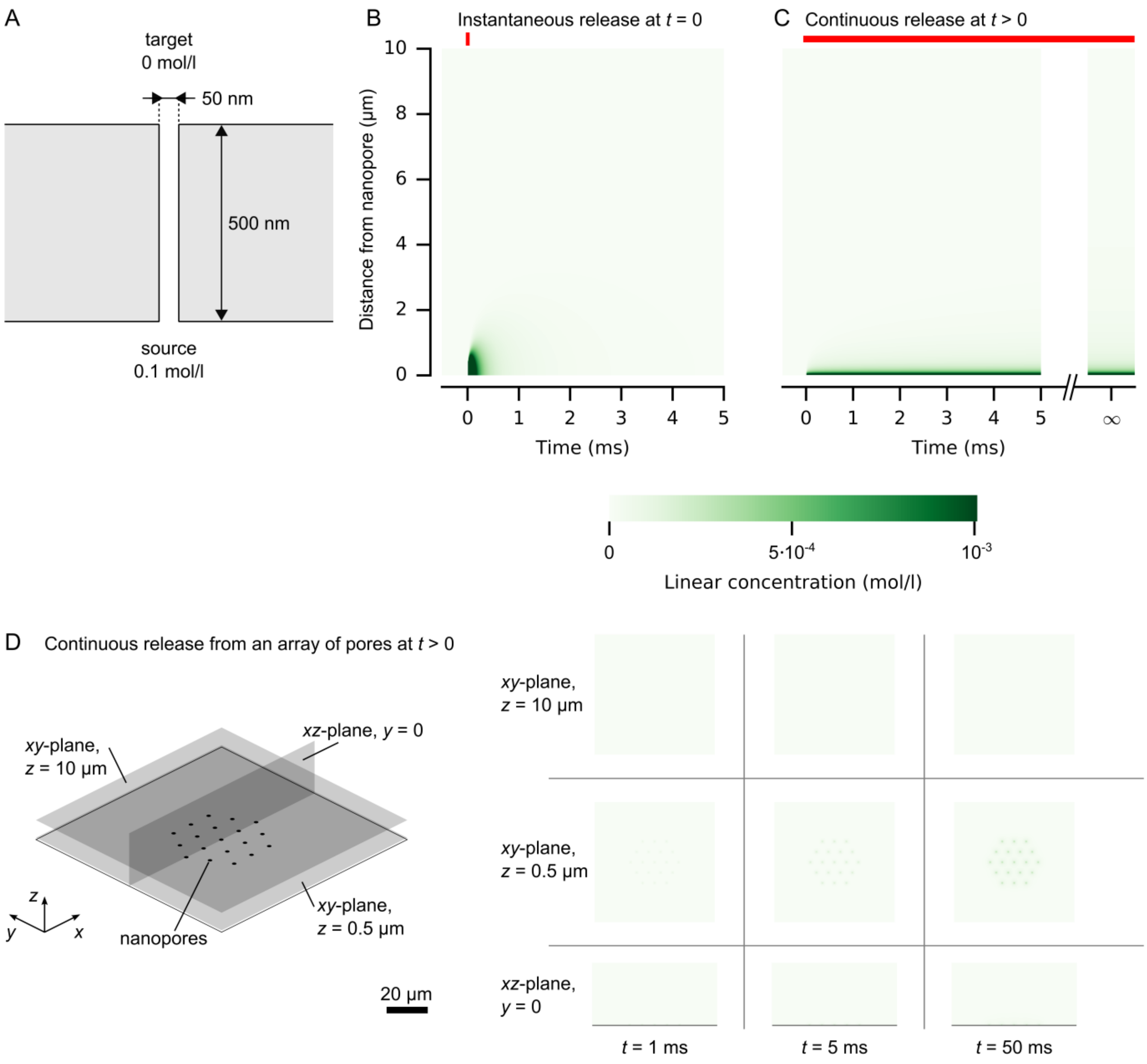

Figure 1S: Release and spread of chemical signals from nanopores by diffusion (linear color scale). (A) The physical setup of a single nanopore with diameter of 50 nm and length of 500 nm. (B) Propagation of an instantaneous chemical impulse of 10^6 molecules at $t = 0$. Concentrations 1 µm away from the nanopore source rise and fall by orders of magnitudes within milliseconds. (C) Propagation of constant release from a nanopore turned on at $t > 0$. A high concentration is established quickly near the nanopore. At larger distances, the concentration approaches a steady state, which is diluted by orders of magnitude in comparison to the concentration within 1 µm of the nanopore. (D) A dense array of point sources can be resolved at close distances. Here, the release rate at each nanopore is the same as in (C) and the nanopores are separated by 10 µm.

4 References

Babakinejad, B., Jönsson, P., López Córdoba, A., Actis, P., Novak, P., Takahashi, Y., et al. (2013).

Local delivery of molecules from a nanopipette for quantitative receptor mapping on live cells. *Anal. Chem.* 85, 9333–42. doi:10.1021/ac4021769.

Cottin-Bizonne, C., Cross, B., Steinberger, A., and Charlaix, E. (2005). Boundary slip on smooth hydrophobic surfaces: Intrinsic effects and possible artifacts. *Phys. Rev. Lett.* 94, 1–4. doi:10.1103/PhysRevLett.94.056102.

Crank, J. (1975). *The mathematics of diffusion*. 2nd ed. London: Oxford University Press.

Hepburn, I., Chen, W., Wils, S., and De Schutter, E. (2012). STEPS: efficient simulation of stochastic reaction-diffusion models in realistic morphologies. *BMC Syst. Biol.* 6, 36. doi:10.1186/1752-0509-6-36.

Herr, N. R., Kile, B. M., Carelli, R. M., and Wightman, R. M. (2008). Electroosmotic flow and its contribution to iontophoretic delivery. *Anal. Chem.* 80, 8635–8641. doi:10.1021/ac801547a.

Syková, E., and Nicholson, C. (2008). Diffusion in brain extracellular space. *Physiol. Rev.* 88, 1277–1340. doi:10.1152/physrev.00027.2007.

Tritton, D. J. (1988). *Physical Fluid Dynamics*. 2nd ed. Clarendon Press.
