# Supplementary figures and images for: Can Nanofluidic Chemical Release Enable Fast, High Resolution Neurotransmitter-Based Neurostimulation?
Source: Front Neurosci. 2016 Mar 31;10:138. doi: 10.3389/fnins.2016.00138 (PMC4815362; doi:10.3389/fnins.2016.00138)

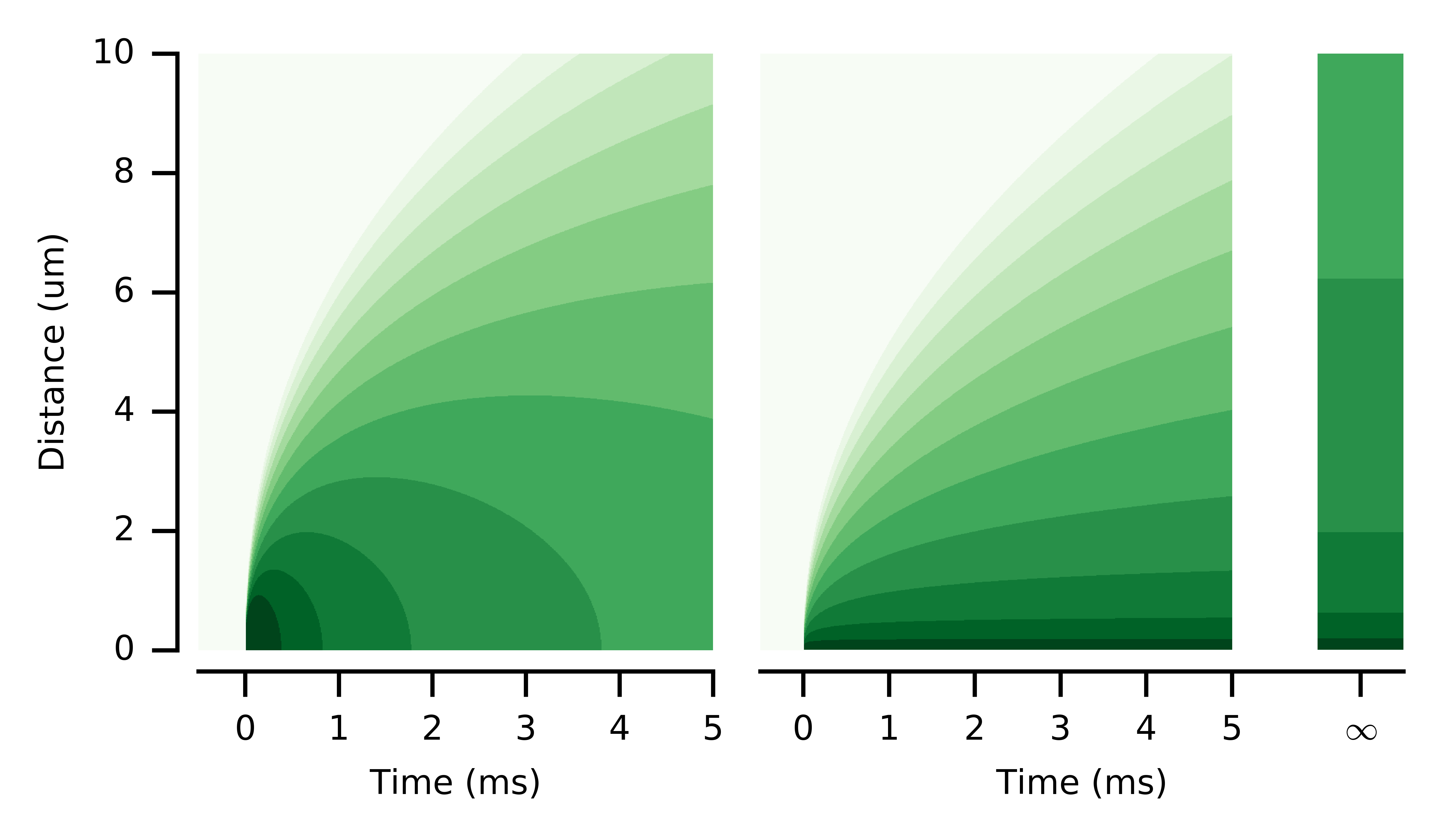

Supplement: Supplementary file 2 [file DataSheet2.ZIP › figures/ChemicalSignalPropagation.png]

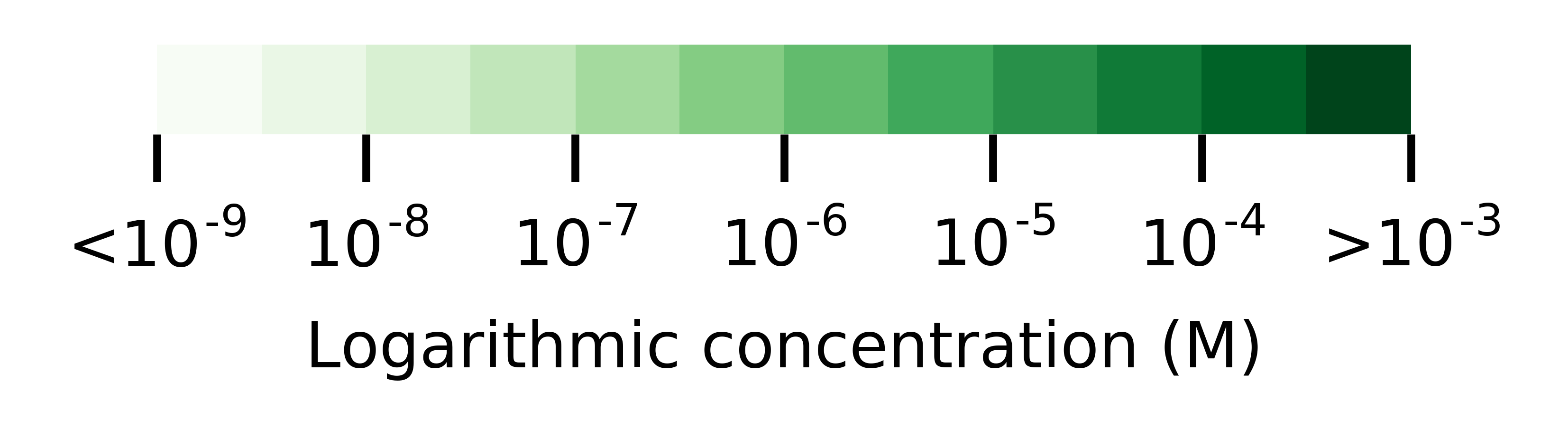

Supplement: Supplementary file 2 [file DataSheet2.ZIP › figures/colorbar_horiz.png]

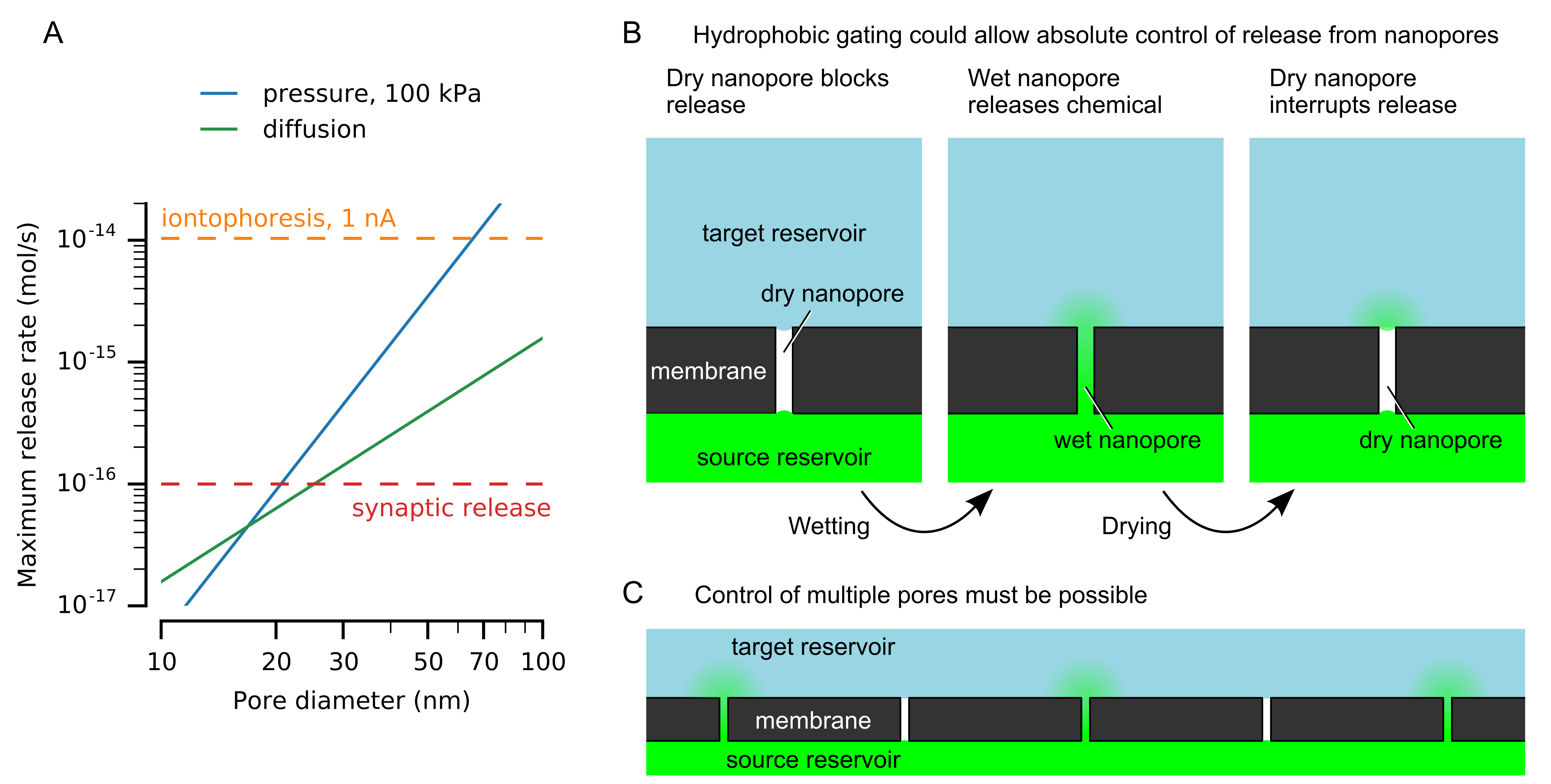

Supplement: Supplementary file 2 [file DataSheet2.ZIP › figures/Figure1.png]

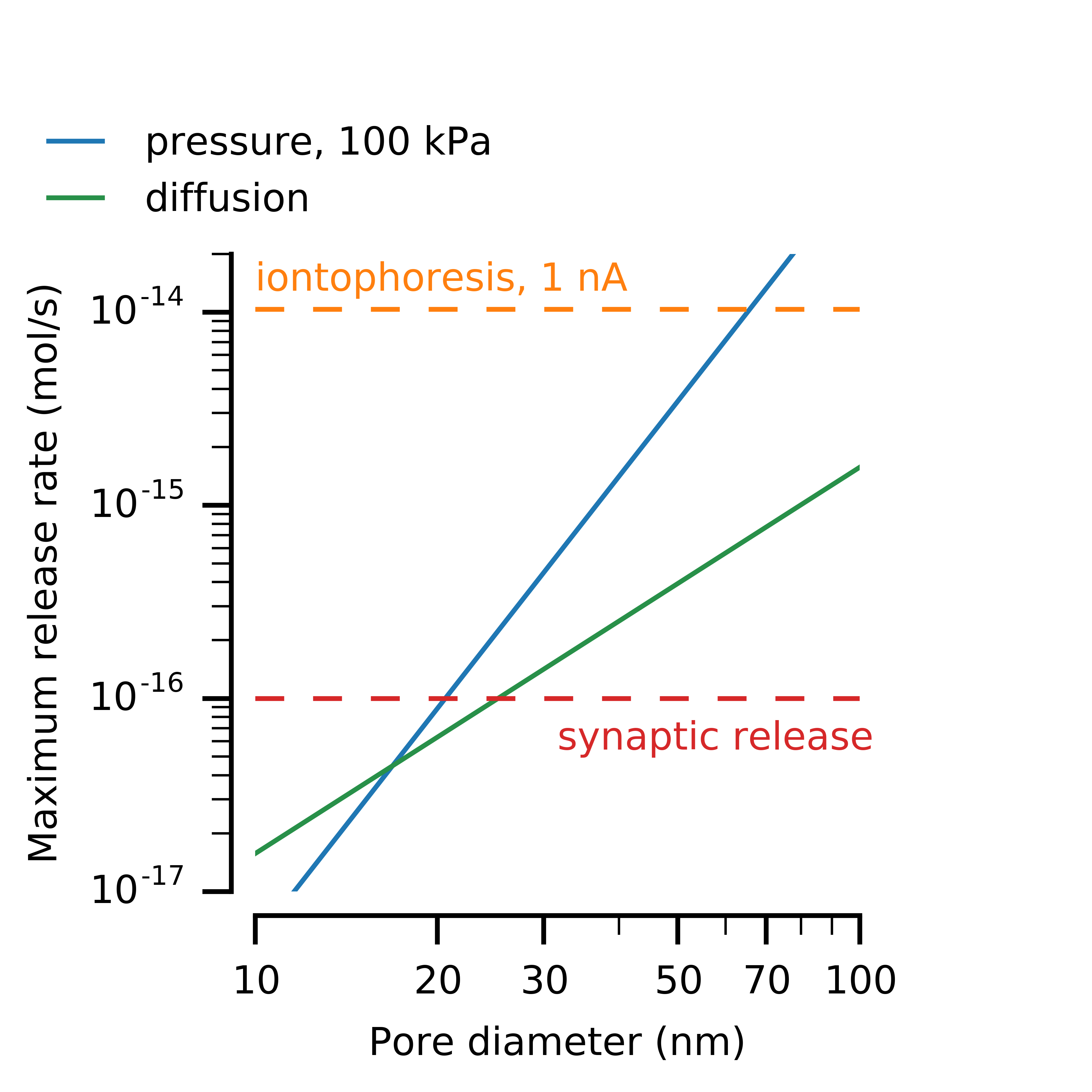

Supplement: Supplementary file 2 [file DataSheet2.ZIP › figures/NanoporeReleaseRates.png]

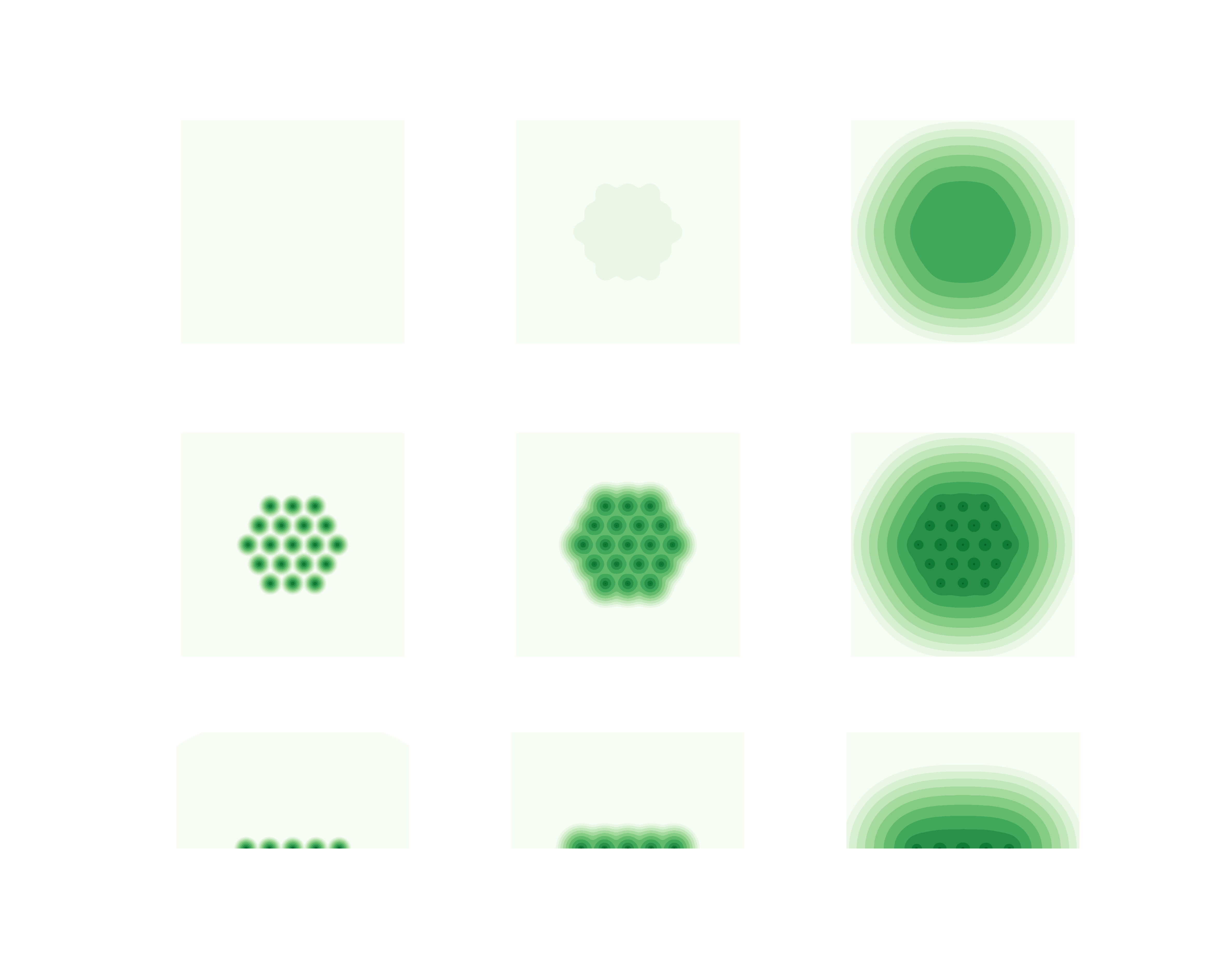

Supplement: Supplementary file 2 [file DataSheet2.ZIP › figures/xyzt.png]
